# Advantages of learning objectives and the National Competence-based Catalogue of Learning Objectives

**DOI:** 10.3205/zma001647

**Published:** 2023-09-15

**Authors:** Marjo Wijnen-Meijer

**Affiliations:** 1 Technical University of Munich, School of Medicine, TUM Medical Education Center, Munich, Germany

## Editorial

In Germany the improvement of medical education is the basis to ensure a high level of health care. The education of physicians is regulated by the state. The legal basis are the Medical Licensing Regulations [1]. 

The National Competence-based Catalogue of Learning Objectives in Medicine 2.0 (NKLM) released in 2021 especially focuses on physician-patient communication and will be mandatory for all German medical faculties [2]. It is including but not limited designed to directly improve care and takes effect as soon as the new Medical Licensing Regulations become operative [1], [3]. 

Learning target indexes represent a first step to further improve medical education due to all topics remain sufficiently represented despite the specification. It is necessary that new teaching standards are practiced particularly with regard to the topics and learning targets of the NKLM 2.0 due to the National Competence-based Catalogue of Learning Objectives in Medicine will be mandatory in the future. It will become the basis to improve educational standards, but not exclusively, to make a contribution to the curriculum development [1]. 

The fundamental idea is to increase patient safety and to intercede all responsibilities needed to become a good medical doctor [4]. Several domains of medical education like basic sciences, clinical knowledge, ethical principles and competencies in knowledge, emotions, communication, reflection and practical skills are included [5]. This should constitute the basis for further steps of teaching contents. But there is scope for development in terms of learning content, so intra-faculty as well as inter-faculty discussions of the curriculum are advisable [1]. An essential component of the National Competence-based Catalogue of Learning Objectives in Medicine is to guarantee a combination between theory and practice as well as to describe all skills which medical students have to have at their disposal at the end of their medical studies [6]. Therefore it can be used as a guide for teaching inter- and multi-professionally in medical studies.

Learning objectives which are specified by the German Council of Medical Faculties and the German Medical Association focus on the purpose of medical education to enhance the learning experience for students and for the academic field and the comparability of educational quality in preparation to an outstanding postgraduate medical qualification [5]. 

Since the 1960s the decline in physical examination proficiency is occurring [7]. New, modern, forward-looking training strategies are requested for the medical profession of tomorrow. Therefore the most important point is to figure the junior physicians’ competencies out and make sure that the core curriculum creates a comprehensive and standardized medical education with a structured outline of the units [8]. So competency-based medical education formats get part of the medical studies as well as new examination formats will substitute the current multiple-choice-question format [3]. 

University teachers should use learning objectives as a basis for teaching units to promote the learning success of the students [9]. So the consequence should be that a connection between what is taught, learned and tested is ensured, which is called “constructive alignment”. In addition it should describe the skills of the students after completing a teaching unit [10]. For the learning process interactive learning strategies are important to improve the desired results as well as to disclose medical mistakes and to determine an adverse event [11]. 

Learning objectives engender to structure lessons on a rational and scientific basis and to compile curricula and appropriate exams [12]. Further they should establish a basis of a module [9]. It is recommended to phrase them specific, measurable, attainable, relevant, and time-bound (SMART) in consideration of Benjamin Bloom’s Taxonomy, which describes the categorizing of educational goals [13]. It is a framework consisting of six levels of cognitive thinking usually illustrated as a pyramid. Since 2001 an adapted version describes the verbs of these levels in six major categories – remember, understand, apply, analyze, evaluate and create. The basis for these processes is profound knowledge [14]. 

Learning objectives define responsibilities of students and teachers to achieve the desired learning outcome and a focused mindset for students for a more rich and challenging learning experience. Students, assessment offices, accreditors, instructional designers and faculties use learning objectives [15]. 

In summary learning objectives act as guideposts for both, students and professors, furthermore they promote structured teaching strategies and systematic learning for the students, avoid unwanted redundancies in the curriculum, are designed to improve the quality and transparency of education for students as well as for teachers and facilitate the compilation of examination questions. So the increasing of teaching-quality could make a meaningful contribution to professionalizing in an interdisciplinary context [6]. 

Learning objectives play an implicit or explicit role in many articles in this issue. When developing and evaluating new learning formats, the starting point is what students need to learn in the particular course. Götz’s planspiel to teach medical-sociological themes is an excellent example [16]. Darici et al. describe how eye-tracking develops students’ visual expertise during an online histology training course [17]. The article by Besse et al. also describes the extent to which students meet the learning objectives – in this case with regard to the theme of trans identity [18]. Other articles examine the effect of different learning formats on achieving learning objectives. Examples include the article by Awad et al. which compares commercial synthetic skin substitutes for surgival simulation training, and Weigel et al. who describe whether students acquire more radiology knowledge in tablet-based or presentation-based seminars [19], [20].

## Competing interests

The author declares that she has no competing interests.
